# The influence of two forms of chlorhexidine on the accuracy of contemporary electronic apex locators

**DOI:** 10.1186/s12903-019-0994-z

**Published:** 2019-12-31

**Authors:** Ewa Marek, Ryta Łagocka, Katarzyna Kot, Krzysztof Woźniak, Mariusz Lipski

**Affiliations:** 1 Department of Preclinical Conservative Dentistry and Preclinical Endodontics, Pomeranian Medical University, Al. Powstańców Wielkopolskich 72, 70-111 Szczecin, Poland; 2 Department of Conservative Dentistry, Pomeranian Medical University, Al. Powstańców Wielkopolskich 72, 70-111 Szczecin, Poland; 3 Department of Orthodontics, Pomeranian Medical University, Al. Powstańców Wielkopolskich 72, 70-111 Szczecin, Poland

**Keywords:** Electronic apex locator, Chlorhexidine digluconate, Sodium hypochlorite, Length determination

## Abstract

**Background:**

Accurate determination of working length (WL) is crucial for the success of endodontic therapy. The aim of this study was to determine the influence of 2% chlorhexidine digluconate solution, 2% chlorhexidine digluconate gel and 2% hypochlorite solution on the accuracy of two devices: the Raypex 5 and the ApexDal.

**Methods:**

Twenty-nine single-rooted human teeth were used in this study. The crowns were cut horizontally and embedded in an alginate mass. In each tooth, six endodontic measurements were made using two electronic apex locators (EALs): a Raypex 5 and an ApexDal. For each EAL, measurements were taken with the following products: 2% chlorhexidine solution (CHX-S group), 2% chlorhexidine gel (CHX-G group) and 2% NaOCl (NaOCl group). After performing an endodontic measurement, the endodontic instruments were stabilized with flow resin composite. Afterwards, the roots were removed from the alginate mass, and the apical one-third of each root was cut lengthways to recover the canal system. Last, the distance between the file tip and the apical foramen was measured under a microscope at 60 x magnification.

**Results:**

Statistically significant differences were found between CHX-S and NaOCl and CHX-G and NaOCl, but no significant differences were detected between CHX-S and CHX-G during the testing of both devices. No statistically significant differences were observed between the Raypex 5 and ApexDal for all intracanal media tested.

**Conclusion:**

The EALs Raypex 5 and ApexDal had higher accuracy in the anatomical foramen of the root containing chlorhexidine in the gel or in the solution form than in the canal containing sodium hypochlorite.

## Background

Accurate working length (WL) determination is a challenge for dentists [1]. The proper point at which root canals should be prepared and obturated is the cementodentinal junction (CDJ), which is also described as the minor foramen. This is the area where the pulp ends and the periodontal ligament begins. Stopping endodontic treatment in the apical constriction saves the mixed tissues, which have regenerative and metaplastic abilities that provide a barrier formation that protects the periodontium [2]. The apical constriction is also the narrowest portion of the canal in which pulp should be cut, creating the smallest possible wound, and thus the periodontium is not damaged and the conditions for healing are the best [3, 4]. Traditional methods for locating the apical constriction include radiography (digital-tactile sense), tactile sensation or paper point techniques. All of these methods have limitations [5]. The apical vertex viewed on a radiograph does not always coincide with the CDJ, minor or major foramen [6]. Over time, secondary dentin and cementum change the position of the apical constriction, causing more preparation errors. When the apical foramen exits in a buccal or lingual direction, it is very difficult to determine the WL [7]. Additionally, anatomical structures can obscure the apex of the root. For example, the zygomatic arch can cover the roots of the upper molars. In addition, the radiograph can only provide a two-dimensional image of a three-dimensional object [8–10]. Additionally, the paper point technique is only an empirical method [11]. With these techniques, canals should be completely dry, and the periapical tissues should be relatively moist. These problems have been eliminated by using electronic apex locators (EALs) because their readings are not related to the apical vertex but rather the apical constriction [6]. Most electronic measuring devices are based on the theory of Suzuki, who determined that electrical resistance between the oral mucosa and the periodontal tissues was constant at 6.5 kΩ and was independent of the patient’s age or tooth morphology. Sunada, tapping this theory, built the first EAL, which unfortunately gave inaccurate readings in the presence of electrolytes or vital pulp tissues [12]. Later, devices became more sophisticated and have used the characteristics of impedance gradients and frequency dependency to provide more accurate and reliable measurements under typical clinical conditions [13]. These new EALs, in which the problems with canal moisture have been solved, include qualified second-generation apex locators that use single frequencies, third-generation apex locators that use multiple frequencies, fourth-generation apex locators that use two separate frequencies and fifth and sixth generation devices that use multiple frequencies to locate the minor foramen [6]. Such generations of apex locators are, for example, the Raypex 5 (VDV, Germany) and the ApexDal (Septodont, France). The Raypex 5 is a new generation EAL that uses two separate frequencies (400 Hz and 800 Hz) [14]. This device makes use of the same frequencies of alternating currents but bases the measurement on the mean square root values of the electric signals [14]. The ApexDal is a new digital apex locator. It also uses two separate frequency. According to the manufacturer’s instructions, these devices can operate in any root canal environment, including in the presence of moisture, blood, pus, vital and necrotic pulp. Raypex 5 EAL is well tested and trustworthy one [14–19] while there is no studies about ApexDal EAL.

Irrigation is presently the best method for the removal of tissue remnants and dentine debris during instrumentation [20]. All over the years, many materials have been used to the root canal irrigation, and certainly, the sodium hypochlorite (NaOCl), ethylenediaminetetraacetic acid (EDTA) and chlorhexidine gluconate (CHX) are the most popular solutions used and most reliable ones. Due to their wide spectrum antimicrobial activity, an irrigation regimen has been proposed, in which NaOCl would be used throughout instrumentation, followed by EDTA, and CHX would be used as a final irrigant. The combination of NaOCl and CHX has been advocated to enhance their antimicrobial properties [21–24].

Sodium hypochlorite (NaOCl) and chlorhexidine are the most popular irrigation solutions used. Chlorhexidine gluconate (CHX) can replace NaOCl during endodontic treatment [25]. CHX has several advantages, such as its low toxicity, broad antibacterial spectrum, effectiveness against *Enterococcus faecalis* and *Candida albicans* [26–29], substantivity [30], tolerable odor and taste and nonbleaching properties [21]. The concentration of CHX used in endodontics varies from 0.2 to 2%. Recently, CHX in gel form was introduced for endodontic treatment. This gel form is a biocompatible and water-soluble agent that is usually used as an intracanal dressing in infected teeth. The gel can also be used as a lubricant, increasing the mechanical removal of organic tissues and decreasing smear layer formation [31, 32]. The effect of the irrigation solutions as factors potentially affecting EAL accuracy has been studied widely. Study results overwhelmingly describe the lack of influence of the content of the root canal on the results of the measurements [33–37]. However, current research has shown that the accuracy of endometric measurements might depend on the type of irrigation solution used [32, 38–40]. Among irrigation solutions, chlorhexidine digluconate showed the highest accuracy in determining the endodontic WL [38, 39]. To date, only one study has evaluated the effects of CHX gel on EAL accuracy [32].

The aim of this study was to determine the influence of 2% chlorhexidine digluconate solution (CHX-S), 2% chlorhexidine digluconate gel (CHX-G) and 2% NaOCl solution on the accuracy of two devices: the Raypex 5 and the ApexDal. The null hypotheses tested were as follows: (i) the accuracy of the contemporary EAL measurements would not depend on the kind of solution used, and (ii) the accuracy of the contemporary EAL measurements would not depend on the kind of equipment used.

## Methods

Twenty-nine single-rooted vital human teeth (incisors and upper second premolars) that had been scheduled for extraction from patients for periodontal or prosthetic reasons were selected. The patients were aged 35–55 years. The selected teeth were tested with an Analytic Technology pulp tester (Analytic Sybron Dental Specialties, Orange, California, USA) to confirm that they contained vital pulp tissue. Immediately after extraction, the teeth were placed in a 10% formalin solution for 48 h. After fixation, the teeth were stored in 2.5% NaOCl solution for 48 h, and the root surfaces were cleaned to remove all organic debris and deposits. The mesiodistal and buccolingual radiographs were taken to determine that the selected teeth had noncomplicated root canal anatomy, single straight root canals and mature root formation. All roots were inspected under an operating microscope at 12.5x magnification (SmartOPTIC ERGO, Seliga) to determine any sign of external resorption, cracks or fractures along the roots. The research was carried out with the consent of the Ethics Committee of Pomeranian University of Medicine (approval number KB-0012/184/08/19) and was conducted in accordance with the Declaration of Helsinki ethical principles. To take part in this research, all 29 study participants signed a voluntary written consent form (KB- 0012/10/19).

### Teeth root preparation

First, the crowns were cut horizontally with a high-speed diamond flame bur REF F 0250 343, no 16 (Dentsply, Maillefer, Ballaigues, Switzerland) at 2-mm coronal to the CEJ. Then, on the flat occlusal surfaces, edges were made as reproducible reference points. Special edges resembling a cube were made with a round diamond bur REF F 0001 343, no 18 (Dentsply Maillefer, Ballaigues, Switzerland) placed in a turbine. Afterwards, the orifice and 1/3 of the coronal part of each canal were flared with Gates-Glidden drills (sizes 2 to 4) (Mani, Tochigi, Japan). The patency of the canals was checked by inserting of a size 10 K-file (Dentsply Maillefer, Ballaigues, Switzerland) until the tip was visible at the apical foramen.

### Root canal length measurements

**Fig. 1:**
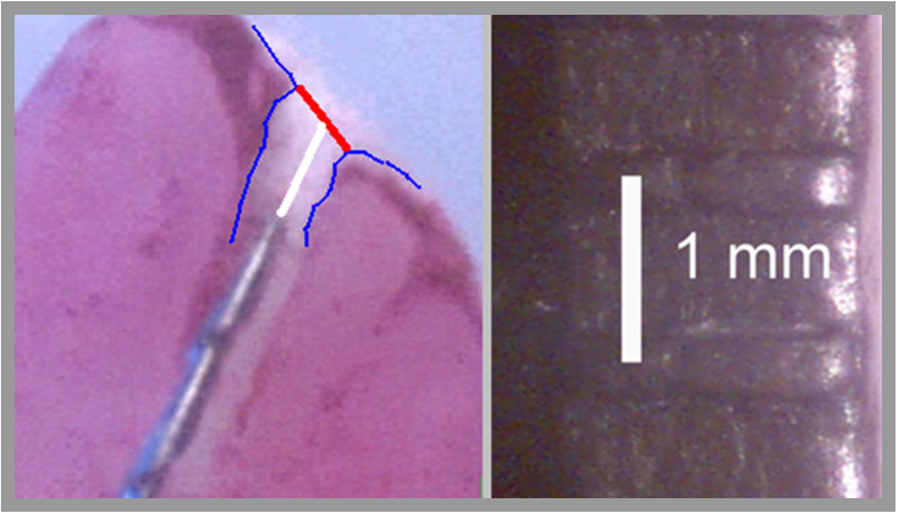
Measurement of the distance between the tip of the file and the anatomical foramen (red line). On the right side a photo of the endodontic ruler. Both the photo of the root apex and the ruler were made in 60x magnification

The teeth were embedded up to the CEJ in an alginate mass (Kromopan, Lascod, Illinois, USA), which was prepared according to the manufacturer’s instructions and poured into a plastic container. A metal lip clip was also placed into the alginate mass to close the current circulation. The measurements were always performed in the moist alginate mass, according to the model developed by Kaufman and Katz, i.e., within 15–20 min for one tooth. When not in use, the container with the alginate mass was wrapped with wet paper and refrigerated [41]. Measurement were made using two electronic apex locators: Raypex 5 (VDW, Munich, Germany) and ApexDal (Septodont, Saint-Maur-des-Fossés, France). Each EAL was used according to manufacturers’ recommendations for detecting the major apical foramen. For Raypex 5, on the right side of this display has been placed segments (apex zoom) which fill-up while the file advances along the apical region. In the study, the file was advanced within the root canal until the device indicated a red segment on the screen of display. Red segment indicate that the apical foramen has been reached. ApexDal has 8 LEDs to mark the advancement of the file, green diode, next to the last diode indicate apical foramen, additionally indicated as “APEX” reading. For each EAL, measurements were taken with the following products: 2% chlorhexidine solution (Gluxodent, CHEMA, Poland) (group CHX-S), 2% chlorhexidine gel (Gluxogel, CHEMA, Poland) and 2% NaOCl (Chloran, CHEMA, Poland) (group NaOCl). Six endodontic measurements were taken for each tooth: two measurements in a presence of 2% NaOCl; one measurement using Raypex 5 and one measurement using ApexDal, another two measurements in a 2% CHX solution environment using Raypex 5 and ApexDal and two measurements in a 2% CHX gel environment with both devices respectively. Irrigants were placed into the canal using an irrigation syringe and needle 0,3 mm (30ga) (Appli-Vac Irrigating Needle Tip; Vista Dental, Racine, WI, USA). Each canal was filled with 2 mL of irrigant, and the excess fluid was drained with a cotton pellet. Chlorhexidine gel was introduced directly from syringe using needle 0,3 mm (30ga) (Appli-Vac Irrigating Needle Tip; Vista Dental, Racine, WI, USA), the apical part of the canal was filled with chlorhexidine gel using #10 K-file in counterclockwise movement. Each canal was filled with 0,2 mL of gel. The canals were thoroughly rinsed for 2 minutes with 10 mL distilled water and dried with paper points before application of another solution or gel. All measurements were made directly after placement the irrigant or gel into the canal. For every tooth, a new preparation of alginate mass was made. After performing an endometric measurement, the endodontic instrument was stabilized with flow resin composite (Tetric Flow, Ivoclar Vivadent, Lichtenstein) without using a bonding agent. The composite coated the occlusal surface of the tooth and the file up to the shank of the file and was polymerized in this position. Then, the file was uncemented, and another measurement was taken with the new file in the same root. Measurements were performed until all irrigants and the gel were tested with both devices. In total, 6 measurements were taken in each tooth. During decementing of the file, the tooth remained in the alginate mass. Afterwards, the roots were removed from the alginate mass, and the apical one-third of each root was cut lengthways to recover the canal system. Such prepared roots were stained with pioctanine dye for visualization of the anatomical details. Using a microscope (Intel Play QX3 Microscope, USA), pictures of the files being inserted into the canals were taken at 60 x magnification. Last, the distance between the file tip and apical foramen was also measured under the microscope at 60 x magnification. To take solid measurements using every photograph, without the roots, an endodontic ruler was placed with a visible millimeter scale (Fig. 1). Since 29 human teeth were used in the examination, 174 measurements were taken.

### Statistical analysis

Statistical analysis was performed using STATISTICA for Windows 9.0 (StatSoft, Inc.). To evaluate differences between values, the following nonparametric tests were used: Friedman’s ANOVA, Wilcoxon’s matched-pair test and chi^2^ test. A probability of less than 0.05 was considered significant.

## Results

Table 1 presents the measured distance from the tip of the file relative to the apical foramen in different solutions using the Raypex 5 EAL. It is worth noting the longest distance in the NaOCl solution compared with both CHX solutions. Statistically significant differences (*p* < 0.05) were found between CHX-S and NaOCl and CHX-G and NaOCl, but no significant difference was detected between CHX-S and CHX-G (Table 1).

**Table 1:** Distance from the tip of file relative to the apical foramen using Raypex 5 EAL

Group	Mean (mm)	SD (mm)	Minimum (mm)	Maximum (mm)
CHX-S	0.34	0.30	0.0	1.38
CHX-G	0.28	0.23	0.0	0.69
NaOCl	0.55	0.34	0.0	1.61

*CHX-S* group in which canals were irrigated with 2% chlorhexidine solution, *CHX-G* group in which canals were irrigated with 2% chlorhexidine gel, *NaOCl* group in which canals were irrigated with 2% hypochlorite solution, *SD* Standard Deviation

The apical foramen (±0.5 mm) was located 79.3% of the time for CHX-S, 86.2% of the time for CHX-G and 53.2% of the time for NaOCl. There was no significant difference between CHX-S and CHX-G, but there were differences when CHX-S or CHX-G were compared with NaOCl (Table 2).

**Table 2:** File tip position to the major foramen using Raypex 5 EAL

Distance from apical foramen (mm)	CHX-S		CHX-G		NaOCl	
	(*n* = 29)	%	(*n* = 29)	%	(*n* = 29)	%
> − 1	1	3,45	0	0	3	10.34
-1 to – 0.51	5	17.24	3	10.34	10	34.5
- 0.5 to 0	21	72.41	25	86.2	16	55.2
0.1 to 0.5	2	6.9	0	0	0	0
0.51 to 1.0	0	0	1	3.45	0	0

Negative value indicates coronal relative to apical foramen; *CHX-S* group in which canals were irrigated with 2% chlorhexidine solution, *CH-G* group in which canals were irrigated with 2% chlorhexidine gel, *NaOCl* group in which canals were irrigated with 2% hypochlorite solution

Table 3 presents the measured distance from the tip of the file relative to the apical foramen in different solutions using the ApexDal EAL. Similar to the Raypex 5 EAL, the longest distance was obtained in the NaOCl solution. Statistical analysis showed that there were significant differences (*p* < 0.05) between CHX-S and NaOCl as well as CHX-G and NaOCl, but no significant difference was found between CHX-S and CHX-G (Table 3).

**Table 3:** Distance from the tip of file relative to the apical foramen using ApexDal EAL

Group	Mean (mm)	SD (mm)	Minimum (mm)	Maximum (mm)
CHX-S	0.33	0.22	0.0	0.92
CHX-G	0.24	0.21	0.0	0.62
NaOCl	0.64	0.54	0.0	2.07

*CHX-S* group in which canals were irrigated with 2% chlorhexidine solution, *CHX-G* group in which canals were irrigated with 2% chlorhexidine gel, *NaOCl* group in which canals were irrigated with 2% hypochlorite solution, *SD* Standard Deviation

The accuracy of the ApexDal with CHX-S, CHX-G and NaOCl in locating the apical foramen to within ±0.5 mm was 79.3, 86.2, and 48.3%, respectively. There was no significant difference between CHX-S and CHX-G, but there were differences when CHX-S or CHX-G were compared with NaOCl (Table 4).

**Table 4:** File tip position to the major foramen using ApexDal EAL

Distance from apical foramen (mm)	CHX-S		CHX-G		NaOCl	
	(*n* = 29)	%	(*n* = 29)	%	(*n* = 29)	%
> − 1	0	0	0	0	7	24.1
-1 to - 0.51	6	20.7	4	13.8	8	27.6
−0.5 to 0	23	79.3	24	82.75	13	44.8
0.1 to 0.5	0	0	1	3.45	1	3.45
0.51 to 1.0	0	0	0	0	0	0

*CHX-S* group in which canals were irrigated with 2% chlorhexidine solution, *CHX-G* group in which canals were irrigated with 2% chlorhexidine gel, *NaOCl* group in which canals were irrigated with 2% hypochlorite solution, *SD* Standard Deviation

No statistically significant differences were observed between the Raypex 5 and ApexDal for all intracanal media tested.

**Fig. 2:**
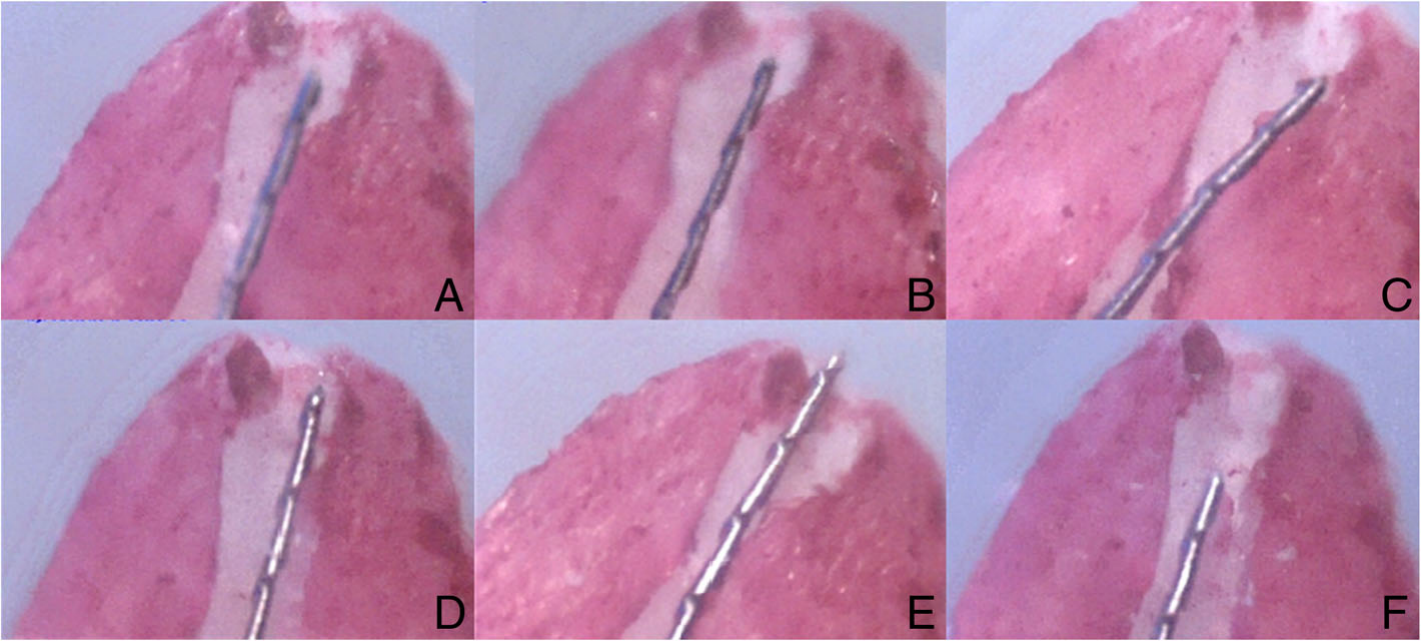
The sample pictures of the apical area of the root with inserted into the canal file. Pictured were taken during measurements with ApexDal (**a**, **b** and **c**) and Raypex 5 EAL (**d**, **e** and **f**) canal at the time were irrigated 2% CHX-S, 2% CHX-G and 2% NaOCl, respectively

Figure 2 shows the sample pictures of the apical area of the root with the canal file inserted. Pictures were taken during measurements with the ApexDal (Fig. 2a, b and c) and Raypex 5 EALs (Fig. 2d, e and f) of canals at the time of irradiation with 2% CHX-S, 2% CHX-G and 2% NaOCl, respectively.

## Discussion

Fifth generation (dual frequency ratio type) EALs measure the resistance and capacitance separately, and there can be different combinations of values of capacitance and resistance that provide the same impedance and thus the same foraminal reading. Therefore, this generation of EAL solved the problem of fourth generation EALs, which must be operated in relatively dry or partially dried canals. The effects of various irrigants, such as saline, hydrogen peroxide, sodium hypochlorite solution, and ethylenediaminetetraacetic acid (EDTA) solution, on fifth generation EAL performance have been investigated. Numerous studies indicate that endodontic measurement can be performed in the presence of any conductive fluid, but the type of irrigant solution may affect the accuracy of the EAL. The most tested irrigants are sodium hypochlorite and chlorhexidine solutions. Erdemir et al. showed no significant difference in file tip position between 2.5% NaOCl and 0.2% chlorhexidine gluconate using a Tri Auto ZX [21]. There were no differences in the accuracy of endometric measurements in the presence of sodium hypochlorite and CHX in the Prasad [42] and Gomes [43] studies. Different results were obtained by Özsezer et al. in an in vivo study; they evaluated the performance of Propex after extirpation and in the presence of different irrigation solutions, including 2.5% NaOCl and 0.2% chlorhexidine gluconate. The results of their study showed that among the irrigation solution groups, the chlorhexidine gluconate group was closest to the actual length [8]. Shin et al. noted that measurements made in the presence of CHX are most consistent [32]. This finding was confirmed in this study. The distance to the apex was smallest when CHX substances were used rather than when NaOCl was used (Table 2., Table 4). Therefore, the first null hypothesis was rejected. This result could be a product of the different electrical conductivities of the solutions used. Electrical conductivity is the ability of different types of matter to conduct an electric current. The electrical conductivity of a material is defined as the ratio of the current per unit cross-sectional area to the electric field producing the current [44]. Electrical conductivity is an intrinsic property of a substance that is dependent not on the amount or shape but on the temperature and chemical composition of the substance. WL measurements tended to be slightly shorter in solutions of higher electrical conductivity, such as NaOCl solutions [37].

The literature review revealed that in accurate EAL measurements, usually 0.1, 0.2, 0.8% [4, 8, 21, 45] CHX solutions were tested. However, more recent studies have evaluated 2% CHX solution and 2% CHX gel. Most authors agree that the CHX concentration for root canal treatment should be 2% [46–48]. Chlorhexidine is a bis-biguanide that has broad-spectrum antimicrobial activity and is active against both gram-positive and gram-negative microbes. Only 2% CHX has a sufficiently high kill rate against the common endodontic pathogen *E. faecalis*. In Dammaschke’s research, 2% CHX-G was as effective as camphorated-and-mentholated chlorophenol (ChKM) against *E. faecalis* [49]. Thus, when choosing a root canal medicament, the established better biocompatibility of CHX compared with that of ChKM should be taken into consideration [49]. CHX-G, also used as an intracanal medicament in other Dammaschke studies, showed good periapical regeneration, suggesting that the gel may be an alternative to calcium hydroxide root canal dressing [50]. It was shown that CHX can be retained in root canal dentin at effective antimicrobial concentrations for up to 12 weeks [30]. Due to the excellent antibacterial properties, especially against *E. faecalis*, and the long-lasting bactericidal effect observed in this study, a 2% concentration of chlorhexidine in solution and gel form was used.

Many studies assessed the accuracy of endometric measurements performed with different equipment. In Jung et al. [4], an in vitro study tested seven different apex locators in the presence of different solutions, including 0.1% chlorhexidine and 5.25% NaOCl. The researchers obtained no statistically significant differences between the tested EALs (Apex Finder 7005, Apit, Bingo-1020, e-Magic Finder, ProPex, Root ZX, and SmarPex.). Oliveira et al. [51] tested five EALs: the Root ZX II, Raypex 6, Apex ID, Propex II, and Propex Pixi. The researchers revealed no difference in accuracy between the evaluated devices. Gurel et al. [15] also tested a Raypex 6 and two other new generations of apex locators Raypex 5, iPex and iPex II. The accuracy of the WL measurement was similar for all devices. Khandewal et al. [14] conducted comparative evaluation of accuracy of Raypex5 and Apex NRG XFR EALs with conventional radiography in ex vivo study. They obtained the same accuracy in determining the WL for both EALs when compared with radiography Saatchi et al. [52] tested Raypex 5 and Root ZX in the presence of blood and also found no significant differences. Al-Hadlaq [53] evaluated the accuracy of the Root ZX and two compact apex locators, the Root ZX mini and Mini Apex Locator, in the presence of different endodontic solutions, including 2% CHX and NaOCl at 5.25, 2.625, and 1% concentrations. The function of all three devices was similar and was not affected by the type of solution. The lack of differences in the accuracy of measurements made with two different apex locators was also confirmed in this study. Between the Apexdal and Raypex 5, no statistically significant differences were observed for all intracanal media tested. Thus, the second null hypothesis was confirmed. All devices tested in the different solutions provided a high percentage of correct measurements. Generally, measurement accuracy is 83% [4], 90% [54] or higher [55]. In this study, good results were achieved only when canals were filled with CHX solution or CHX gel. Measurement accuracy was 79.3 and 86.2%, respectively, for the devices. Measurements that were made in canals irrigated with 2% NaOCl were correct only in 53.2 and 48.2% of cases for Raypex 5 and ApexDal EAL, respectively. Some studies differ in establishing the reference point from which measurement accuracy is determined. Some researchers measure the minor diameter (apical constriction), whereas others measure the major diameter or apical foramen [56]. It is worth noting that EALs are highly accurate in determining the location of the minor constriction, but the mean distance from the file tip to the minor constriction always has a positive value [56, 57]. This means that EALs mostly overestimate the WL. In this research, the file tip was located in a more apical positive relative to the apical constriction. Such a position of the file tip resulted from the fact that measurements were made until the “apex” was marked by the devices. In this research, correct measurements were taken, and the tip of the file was ±0.5 mm within the apical foramen. For both apex locators, the distance to the apex was smallest when CHX solution and CHX gel were used instead of NaOCl. Ebrahim et al. [45] examined the effects of 0.5% NaOCl, 2.5% NaOCl and 0.8% chlorhexidine on the accuracy of a Dentaport ZX in enlarged canals. They found that the Dentaport ZX was accurate and not affected by the presence of both NaOCl concentrations when small and large files were used but was accurate in the presence of CHX only when large files were used. In another in vitro study, Jung et al. [4] tested seven different apex locators in the presence of different solutions, including 0.1% chlorhexidine and 5.25% NaOCl. There were no statistically significant differences between the EALs (Apex Finder 7005, Apit, Bingo-1020, e-Magic Finder, ProPex, Root ZX, and SmarPex.). The same conclusions are presented in this study. Between the ApexDal and Raypex 5, no statistically significant differences were observed for all intracanal media tested. However, the studies by Shin noted that the measurements taken in the presence of CHX were the most consistent [32]. This means that the CHX solution resulted in the least variability in the performance of the devices. This study confirms this observation. There was also no significant difference between CHX-S and CHX-G, but there were differences when CHX-S or CHX-G were compared with 2% NaOCl. Jenkis et al. [37] tested a Root ZX in the presence of 2% lignocaine, 5.25% NaOCl, RC Prep, EDTA and 3% H_2_O_2_. The researchers did not find an influence of the irrigant solution on the performance of the device, but Root ZX worked least precisely in the presence of 5.25% NaOCl. In this study, in addition to 2% NaOCl, different solutions were tested than those in the Jenkis study, but both EALs achieved the poorest results in the presence of NaOCl. Kaufman et al. [58] tested a Root ZX and Bingo 1020. They found significant differences based on the canal conditions; measurements were closer to the actual length in the presence of EDTA and saline than in dried or Xylol-filled canals. Their results also indicated that electric measurements can be safely performed in the presence of CHX, because these results were similar to those obtained in the presence of NaOCl.

## Conclusions

The apex locators Raypex 5 and ApexDal locate with the highest accuracy the anatomical foramen of the root containing chlorhexidine in the gel or in the solution form than in canal containing the sodium hypochlorite.

## Data Availability

The datasets used and/or analysed during the current study are available from the corresponding author on reasonable request.

## Abbreviations

CDJ: Cementodentinal junction
CHX-G: chlorhexidine digluconate gel
CHX-S: Chlorhexidine digluconate solution
EAL: Electronic apex locators
NaOCl: Sodium hypochlorite
WL: Working length

## References

[1] Ziegler AA, Altenburger MJ, Wrbas KT (2007). In vivo comparison of working length determination with electronic apex locators. Int Endod J.

[2] Stein TJ, Corcoran JF (1991). Noioniozing method of locating the apical constriction (minor foramen) in root canals. Oral Surg Oral Med Oral Pathol.

[3] Basmadijan-Charles CL, Farge P, Bourgeois DM, Lebrun T (2002). Factors influencing the long-term results of endodontic treatment: a review of the literature. Int Dent J.

[4] Kang JA, Kim SK (2008). Accuracies of seven different apex locators under various conditions. Oral Surg Oral Med Oral Pathol Oral Radiol Endod.

[5] Plotino G, Grande NM, Brigsnte L, Lesti B, Somma F (2006). Ex vivo accuracy of three electronic apex locators root ZX, elements diagnostic unit, apex locator and ProPex. Int Endod J.

[6] Meares WA, Steiman HR (2002). The influence of sodium hypochlorite irrigation on the accuracy of the root ZX electronic apex locator. J Endod.

[7] Kim E, Marmo M (2008). An in vivo comparison of working length determination by only root ZX apex locator versus combining root ZX apex locator with radiographs using a new impression technique. Oral Surg Oral Med Oral Pathol Oral Radiol Endod.

[8] Ózsezer E, Inan U, Aydin U (2007). In vivo evaluation of ProPex electronic apex locator. J Endod.

[9] ElAyouti A, Weiger R, Löst C (2001). Frequency of overinstrumentation with an acceptable radiographic working length. J Endod.

[10] ElAyouti A, Weiger R, Löst C (2002). The ability of root ZX apex locator to reduce the frequency of overestimated radiographic working length. J Endod.

[11] Marcos-Arenal JL, Rivera EM, Caplan DJ (2009). Evaluation the paper point technique for locating the apical foramen after canal preparation. Oral Surg. Oral Med Oral Pathol Oral Radiol Endod.

[12] Dunlap CA, Remeikis NA, BeGole EA, Rauschenberger CR (1998). An in vivo evaluation of an electric apex locator that uses the ratio method vital and necrotic canals. J Endod.

[13] Pratten DH, McDonald NJ (1996). Comparison of radiographic and electronic working lengths. J Endod.

[14] Khandewal D, Ballal NV, Sarawathi MV (2015). Comparative evaluation of accuracy of 2 electronic apex locators with convetional radiography: an ex vivo study. J Endod.

[15] Gurel MA, Helvacioglu Kivanc B, Ekici A (2017). A comparative assessment of the accuracies of Raypex 5, Raypex 6, iPex and iPex II electronic apex locators: an in vitro study. J Istanb Univ Fac Dent.

[16] Yolagiden M, Ersahan S, Suyun G, Bilgec E, Aydin C (2018). Comparison of four electronic apex locators in detecting working length: an ex vivo study. J Contemp Dent Pract.

[17] Saraswathi V, Kedia A, Purayil TP, Ballal V, Saini A (2016). Comparative evaluation of the accuracy of two electronic apex locators in determining the working length in teeth with simulated apical root resorption: an in vitro study. J Conserv Dent.

[18] Marroquín BB, Fernández CC, Schmidtmann I, Willershausen B, Goldberg F (2014). Accuracy of electronic apex locators to detect root canal perforations with inserted me-tallic posts: an ex vivo study. Head Face Med.

[19] Ding J, Gutmann JL, Fan B, Lu Y, Chen H (2010). Investigation of Apex Locators and Related Morphological. J Endod.

[20] Erdemir A, Eldeniz AU, Ari H, Belli S, Esener T (2007). The influence of irrigation solutions on the accuracy of the electronic apex locator facility in the tri auto ZX handpiece. Int Endod J.

[21] Kuruvilla JR, Kamath MP (1998). Antimicrobial activity of 2.5% sodium hypochlorite and 0.2% chlorhexidine gluconate separately and combined, as endodontic irrigants. J Endod.

[22] Mohammadia Z, Giardinoc L, Palazzid F, Asgarya S (2015). Agonistic and Antagonistic Interactions between Chlorhexidine and Other Endodontic Agents: A Critical Review. Iran Endod J.

[23] Basrani BR, Manek S, Fillery E (2009). Using diazotization to characterize the effect of heat orSodium hypochlorite on 2.0% Chlorhexidine. J Endod.

[24] Khadse P, Kamra A, Banga KS (2014). Effectiveness of various intermediate irrigants for the prevention of precipitate formed by the interaction of sodium hypochlorite and chlorhexidine-an in vitro study. Edodontology.

[25] Wang CS, Arnold RR, Trope M, Teixeira BF (2007). Clinical efficiency of 2% chlorhexidine gel in reducing intracanal bacteria. J Endod.

[26] Filho M, Leonardo M, Silva L, Anibal F, Faccioli L (2002). Inflammatory responses to different endodontic irrigating solutions. Int Endod J.

[27] Paquette L, Legner M, Fillery ED, Friedman S (2007). Antibacterial efficacy of chlorhexidine gluconate intracanal medication in vivo. J Endod.

[28] Siqueira JF, Rôças IN, Lopez HP, Magalhães FAC, Uzeda M (2003). Elimination of *Candida albicans* infection of the radicular dentin by intracanal medications. J Endod.

[29] Portenier I, Waltimo TD, Ørstavik D, Haapasalo M (2006). Killing of enterococcus faecalis by MTAD and chlorhexidine digluconate with or without Cetrimide in the presence or absence of dentine powder or BSA. J Endod.

[30] Rosenthal S, Spangberg L, Safavi K (2004). Chlorhexidine substantivity in root canal dentin. Oral Surg Oral Med Oral Pathol Oral Radiol Endod.

[31] Dametto FR, Ferraz CC, Gomes BP, Zaia AA, Teixeira FB, de Souza-Filho FJ (2005). In vitro assessment of the immediate and prolonged antimicrobial action of chlorhexidine gel as an endodontic irrigant against enterococcus faecalis. Oral Surg Oral Med Oral Pathol Oral Radiol Endod.

[32] Shin HS, Yang WK, Kim M, Ko HJ, Cho KM, Park SH, Kim JW (2012). Accuracy of root ZX in teeth with simulated root perforation in the presence of gel or liquid type endodontic irrigant. Restor Dent Endod.

[33] Tinaz AC, Sevimli LS, Görgül G, Türköz G (2002). The effects of sodium hypochlorite concentrations on the accuracy of an apex locating device. J Endod.

[34] Fouad AF, Rivera EM, Krell KV (1983). Accuracy of the Endex with variation in canal irrigants and foramen sizes. J Endod.

[35] Ebrahim AK, Yoshioka T, Kobayashi C, Suda H (2006). The effects of size, sodium hypochlorite and blood on the accuracy of root ZX apex locator in enlarged root canals: an in vitro study. Aust Dent J.

[36] Akisue E, Poli de Figueiredo JA (2007). Influence of pulp vitality on length determination by using the elements diagnostic unit and apex locator. Oral Surg Oral Med Oral Pathol Oral Radiol Endod.

[37] Jenkins JA, Walker WA, Schindler WG, Flores CM (2001). An in vitro evaluation of the accuracy of the root ZX in the presence of various irrigants. J Endod.

[38] Jain S, Kapur R (2012). Comparative evaluation of accuracy of two electronic apex locators in the presence of various irrigants: An in vitro study. Contemp Clin Dent.

[39] Khursheed I, Bansal R, Bansal T, Singh HP, Yadav M, Reddy KJ (2014). A comparative evaluation of working length with digital radiography and third generation apex locator (ProPex) in the presence of various intracanal irrigants: an in vivo/ex vivo study. Dent Res J.

[40] Paras M, Manjunath V, Manjunath MK (2012). Comparison of accuracy of two electronic apex locators in the presence of various irrigants: an in vitro study. J Conserv Dent.

[41] Kaufman A, Katz A (1993). Realiability of root ZX apex locator texted by an in vitro model. J Endod.

[42] Prasad AB, Harshit S, Aastha SA, Deepak R (2016). An Invitro evaluation of the accuracy of two electronic apex locators to determine working length in the presence of various Irrigants. Ethiop J Health Sci.

[43] Gomes S, Oliver R, Macouzet C, Mercadé M, Roig M, Duran-Sindreu F (2012). In vivo evaluation of the Raypex 5 by using different irrigants. J Endod.

[44] Joesten MD, Hogg JL, Castellion ME (2004). The word of chemistry: essentials.

[45] Ebrahim AK, Wadachi R, Suda H (2007). An in vitro evaluation of the accuracy of Dentaport ZX apex locator in enlarged root canals. Aust Dent J.

[46] Schäfer E, Bossmann K (2001). Antimicrobial efficacy of chloroxylenol and chlorhexidine in the treatment of infected root canals. Am J Dent.

[47] Zehnder M (2006). Root canal irrigants. J Endod.

[48] Zamany A, Safavi K, Spångberg LS (2003). The effect of chlorhexidine as an endodontic disinfectant. Oral Surg Oral Med Oral Pathol Oral Radiol Endod.

[49] Dammaschke T, Jung N, Harks I, Schafer E (2013). The effect of different root canal medicaments on the elimination of enterococcus faecalis ex vivo. Eur J Dent.

[50] Dammaschke T, Schneider U, Stratmann U, Yoo JM, Schäfer E (2005). Effect of root canal dressings on the regeneration of inflamed periapical tissue. Acta Odontol Scand.

[51] Oliveira TN, Vivacqua-Gomes N, Bernardes RA, Vivan RR, MAH D, Vasconcelos BC (2017). Determination of the accuracy of 5 electronic apex locators in the function of different employment protocols. J Endod.

[52] Saatchi M, Aminozarbian MG, Noormohammadi H, Baghaei B (2016). Influence of blood on the accuracy of Raypex 5 and root ZX electronic foramen locators: an in vivo study. Braz Dent J.

[53] Al-Hadlaq SM (2013). Effect of chloroform, orange solvent and eucalyptol on the accuracy of four electronic apex locators. Aust Endod J.

[54] Chopra V, Grover S, Prasad D (2008). In vitro evaluating of the accuracy of two electronic apex locators. J Conserv Dent.

[55] Steffen H, Splieth CH, Behr K (1999). Comparison of measurements obtaining with hand files or the canal leader attached to electronic apex locators: an in vitro study. Int Endod J.

[56] Luigi Cianconi L, Angott V, Felici R, Conte G, Mancin M (2010). Accuracy of Three Electronic Apex Locators Compared with Digital Radiography: An Ex Vivo Study. J Endod.

[57] Mancini M, Palopoli P, Iorio L, Conte G, Ciancon L (2014). Accuracy of an Electronic Apex Locator in the Retreatment of Teeth Obturated with Plastic or Cross-linked Gutta-percha Carrier-based Materials: An Ex Vivo Study. J Endod.

[58] Kaufman AY, Keila S, Yoshpe M (2002). Accuracy of new apex locator: an in vitro study. Int Endod J.

